# Calcium channel modulation as a target in chronic pain control

**DOI:** 10.1111/bph.13789

**Published:** 2017-04-26

**Authors:** Ryan Patel, Carlota Montagut‐Bordas, Anthony H Dickenson

**Affiliations:** ^1^ Department of Neuroscience, Physiology and Pharmacology University College London London UK

## Abstract

Neuropathic pain remains poorly treated for large numbers of patients, and little progress has been made in developing novel classes of analgesics. To redress this issue, ziconotide (Prialt™) was developed and approved as a first‐in‐class synthetic version of ω‐conotoxin MVIIA, a peptide blocker of Ca_v_2.2 channels. Unfortunately, the impracticalities of intrathecal delivery, low therapeutic index and severe neurological side effects associated with ziconotide have restricted its use to exceptional circumstances. Ziconotide exhibits no state or use‐dependent block of Ca_v_2.2 channels; activation state‐dependent blockers were hypothesized to circumvent the side effects of state‐independent blockers by selectively targeting high‐frequency firing of nociceptive neurones in chronic pain states, thus alleviating aberrant pain but not affecting normal sensory transduction. Unfortunately, numerous drugs, including state‐dependent calcium channel blockers, have displayed efficacy in preclinical models but have subsequently been disappointing in clinical trials. In recent years, it has become more widely acknowledged that trans‐aetiological sensory profiles exist amongst chronic pain patients and may indicate similar underlying mechanisms and drug sensitivities. Heterogeneity amongst patients, a reliance on stimulus‐evoked endpoints in preclinical studies and a failure to utilize translatable endpoints, all are likely to have contributed to negative clinical trial results. We provide an overview of how electrophysiological and operant‐based assays provide insight into sensory and affective aspects of pain in animal models and how these may relate to chronic pain patients in order to improve the bench‐to‐bedside translation of calcium channel modulators.

**Linked Articles:**

This article is part of a themed section on Recent Advances in Targeting Ion Channels to Treat Chronic Pain. To view the other articles in this section visit http://onlinelibrary.wiley.com/doi/10.1111/bph.v175.12/issuetoc

**Abbreviations**
CPM: conditioned pain modulation
CPP: conditioned place preference
CRMP2: collapsin response mediator protein 2
DRG: dorsal root ganglion
NNT: number needed to treat
NS: nociceptive specific
SNL: spinal nerve ligation
VGCC: voltage‐gated calcium channel
VPL: ventral posterolateral
WDR: wide dynamic range

## Introduction

Neuropathic syndromes are often characterized by a complex combination of positive and negative sensory phenomena including allodynia (perceiving non‐noxious stimuli as pain), hyperalgesia (increased pain to normally painful stimuli) and also paroxysmal or persistent paraesthesias (abnormal sensations, e.g. numbness and tingling) and dysaesthesias (abnormal and unpleasant sensations, e.g. shocking and paradoxical burning). These symptoms are often confounded by co‐morbidities such as depression, anxiety and sleep disturbances. Meta‐analyses suggest that pain with neuropathic characteristics has a prevalence of 7–10% in the general population (van Hecke *et al.,* 2014) and that large numbers of neuropathic patients fail to achieve adequate relief from currently available treatments (Finnerup *et al.,* 2010; Finnerup *et al.,* 2015). These studies highlight the urgent need for novel classes of therapeutics and better utilization of currently available treatments. In an attempt to address this ongoing challenge, the first‐in‐class drug http://www.guidetopharmacology.org/GRAC/LigandDisplayForward?ligandId=2536 (Prialt™) was developed as a synthetic version of the http://www.guidetopharmacology.org/GRAC/ObjectDisplayForward?objectId=533 blocker http://www.guidetopharmacology.org/GRAC/LigandDisplayForward?ligandId=2536, derived from *Conus magus*, and is licensed for chronic pain. However, due to the narrow therapeutic window and considerable side effects, ziconotide is only administered intrathecally to patients who have failed to respond to other treatments (Sanford, 2013).

As a rare example of bench‐to‐bedside translation, intrathecal ω‐conotoxins were demonstrated to be anti‐nociceptive in neuropathic animals over 20 years ago (Chaplan *et al.,* 1994). Pain has both sensory and affective dimensions but is also a subjective experience; hence by definition, pain in animals must be inferred. As in this study, most animal behavioural experiments rely on evoked reflex withdrawals to assess ‘pain responses’. A binary outcome measure of this nature renders it near impossible to correlate the intensity of a stimulus with the level of response given that a reflex response determines the first point at which a stimulus becomes aversive. As a consequence, the validity of stimulus‐evoked reflexes as predictors of clinical efficacy has been questioned in light of the fact that many pharmacological agents are efficacious in preclinical models, including calcium channel blockers, but fail to translate into the clinic (Percie du Sert and Rice, 2014). The thorny issue of translation is of course far more complex, and there are numerous instances where choice of appropriate endpoints and identifying underlying mechanisms of neuropathy has led to successful forward and back‐translation. Heterogeneity of patient populations is likely to have led to the failure of many clinical trials and sensory profiling and stratification of patients as an alternate approach to aetiological grouping may lead to better indicators of successful treatment (Baron *et al.,* 2012; Freeman *et al.,* 2014; Bouhassira and Attal, 2016). For example, in diabetic neuropathy, low conditioned pain modulation (CPM) predicts efficacy of http://www.guidetopharmacology.org/GRAC/LigandDisplayForward?ligandId=202 and http://www.guidetopharmacology.org/GRAC/LigandDisplayForward?ligandId=7477 (Yarnitsky *et al.,* 2012; Niesters *et al.,* 2014), and in peripheral neuropathy patients, http://www.guidetopharmacology.org/GRAC/LigandDisplayForward?ligandId=7254 was more efficacious in those with the irritable nociceptor versus the non‐irritable phenotype [number needed to treat (NNT) of 4 and 13 respectively] (Demant *et al.,* 2014). As illustrations of successful back‐translation, in the spinal nerve ligation (SNL) model of neuropathy in rats, CPM or diffuse noxious inhibitory controls are absent and can be restored by http://www.guidetopharmacology.org/GRAC/LigandDisplayForward?ligandId=4808 and tapentadol, and http://www.guidetopharmacology.org/GRAC/LigandDisplayForward?ligandId=5339 inhibits spinal neuronal excitability (Chapman *et al.,* 1998; Bannister *et al.,* 2015). In this respect, the SNL model could represent comparable underlying mechanisms to patients with low CPM or the irritable nociceptor phenotype and thus provides a basis for forward translation. Calcium channel activity could be surmised to be favoured in the SNL model and in patients with the irritable nociceptor phenotype, and so, heterogeneous patient groups based on aetiology only may not provide adequate sensitivity for revealing effects of these drugs.

Most clinical trials continue to rely on ongoing pain scores as a primary clinical endpoint, though more recently in smaller trials, there has been a concerted effort to perform quantitative sensory testing and classify patients according to their sensory profile (Bouhassira and Attal, 2016). This approach attempts to provide a quantitative and qualitative read‐out of the patient's pain state. In terms of a comparable translatable measure, electrophysiological characterizations of neuronal excitability in animals allow reproducible, objective and quantitative measures of sensory neuronal processing to multiple modalities and have the advantage of examining responses to supra‐threshold and brush stimulation which are not particularly amenable to behavioural testing. Ongoing or paroxysmal pain has been difficult to demonstrate in animal models but is a major cause of suffering and poor quality of life in neuropathic patients. As ongoing pain is unpleasant and aversive in its nature, and pain relief is rewarding, a conditioned place preference (CPP) assay in rodents was developed to explore whether injured animals associate with contextual cues affiliated with relief. This method has demonstrated efficacy of clinically used drugs such as intrathecal http://www.guidetopharmacology.org/GRAC/LigandDisplayForward?ligandId=516 (King *et al.,* 2009).

Calcium channel blockers have been successfully developed for the treatment of absence seizures and are an emerging drug class for the treatment of neurological disorders and chronic pain (reviewed in detail by Zamponi, 2016). In this review, we provide an updated overview of the effects of calcium channel blockers in chronic pain models, with a particular focus on how electrophysiological and CPP studies may provide insight into sensory and affective dimensions of pain. Additionally, we consider the prospects of voltage‐gated calcium channels (VGCCs) as targets for chronic pain and how preclinical data may guide design of clinical trials.

## Targeting trafficking of VGCCs

### Auxiliary calcium channel subunit α_2_δ‐1

**Figure 1:**
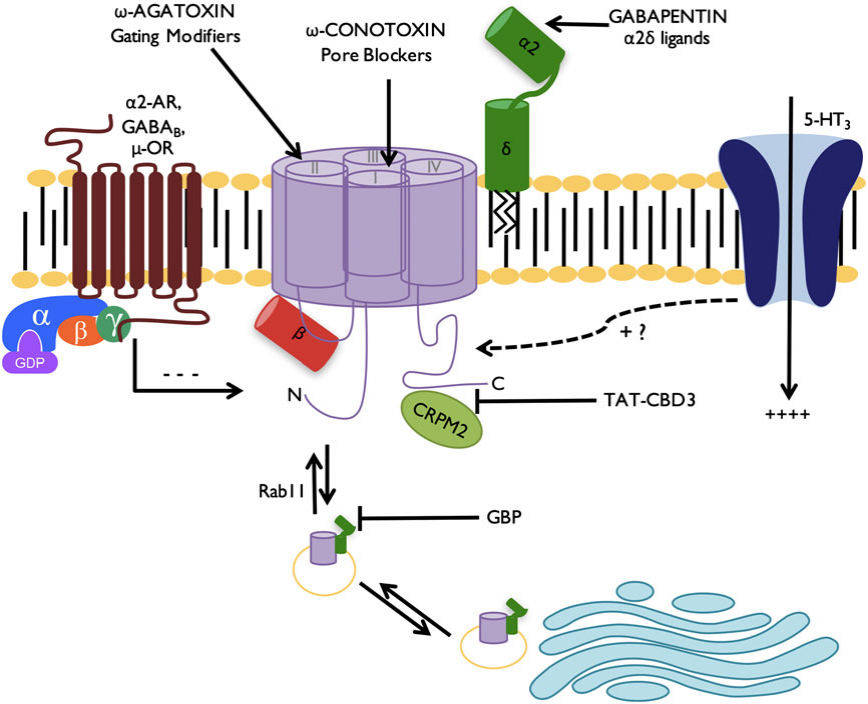
Approaches to calcium channel modulation in chronic pain. To date, targeting trafficking of VGCCs has been the favoured approach to modulating channel activity. The gabapentinoids (GBP) bind to an arginine motif of the α_2_ subunit of α_2_δ‐1 and α_2_δ‐2 and inhibit axonal trafficking of α_2_δ‐1 and Rab11‐dependent recycling of endosomal channels to the synapse (Bauer *et al.,* 2009; Tran‐Van‐Minh and Dolphin, 2010). Novel approaches to reduce trafficking include utilizing CBD3 peptides to disrupt the interaction between VGCCs and CRMP2. Channel activity can be regulated via inhibitory GPCRs such as α_2_ adrenoreceptors, http://www.guidetopharmacology.org/GRAC/FamilyDisplayForward?familyId=26 and μ‐opioid receptors. Peptide blockers act by either altering gating by targeting voltage sensitive domains (such as ω‐agatoxin) or by directly blocking the pore (ω‐conotoxins) (Bourinet and Zamponi, 2016).

By far and away, the most frequently prescribed calcium channel modulators for neuropathic pain are the gabapentinoids, gabapentin and pregabalin. http://www.guidetopharmacology.org/GRAC/LigandDisplayForward?ligandId=5483 and the newer derivative http://www.guidetopharmacology.org/GRAC/LigandDisplayForward?ligandId=5484 were designed as analogues of http://www.guidetopharmacology.org/GRAC/LigandDisplayForward?ligandId=1067. As it transpired, gabapentin was identified as a ligand for the auxiliary voltage‐gated calcium channel subunit α_2_δ rather than GABA receptors (Gee *et al.,* 1996). Transgenic approaches strongly support that the interaction of gabapentinoids with α_2_δ‐1 is required for anti‐nociceptive activity in neuropathic conditions given the lack of efficacy in the corresponding null mice (Field *et al.,* 2006; Patel *et al.,* 2013). At the cellular level, it is unclear precisely how gabapentinoids disrupt transmitter release, but a direct block of channel activation seems highly unlikely (Hendrich *et al.,* 2008). Instead, gabapentin has been shown to inhibit axonal trafficking of α_2_δ‐1, which is elevated in injured primary afferents, and also recycling of calcium channel complexes between intracellular compartments and the synaptic membrane (Bauer *et al.,* 2009; Tran‐Van‐Minh and Dolphin, 2010) (Figure 1). The selective anti‐nociceptive effects in facilitated states imply injury‐induced factors that influence efficacy (reviewed in detail by Patel and Dickenson, 2016). Thus, it would be expected that clinical correlates of these mechanisms would be a determinant of the action of these drugs and so affect their NNT.

In behavioural assays, acute, systemically administered, gabapentin exhibits state‐dependent anti‐nociceptive activity when assessed by changes in withdrawal threshold (Field *et al.,* 1997). Both spinal and supra‐spinal mechanisms are likely to be involved as intrathecal and intracerebroventricular pregabalin increase withdrawal thresholds after peripheral nerve injury (Takeuchi *et al.,* 2007). Likewise, when examined against spinal wide dynamic range (WDR) neuronal responses, gabapentin displays state‐dependent inhibitory activity. Increased descending facilitatory drive is key in shaping the inhibitory effects of acute gabapentin treatment after nerve injury, and remarkably, mimicking enhanced descending facilitation in naïve rats recreates the permissive state for gabapentin to act (Suzuki *et al.,* 2005). Increased activity in this pathway underlies the efficacy of pregabalin in a model of opioid‐induced hyperalgesia where no injury exists (Bannister *et al.,* 2011).

In models where central sensitization is evident, even in the absence of pathology or where neuropathy is not the only component, gabapentin and pregabalin reduce spinal neuronal firing to both mechanical‐ and heat‐evoked responses across low and high intensities of stimulation (Donovan‐Rodriguez *et al.,* 2005; Omori *et al.,* 2009; Bannister *et al.,* 2011; Thakur *et al.,* 2012). Patients with osteoarthritis frequently report pain with neuropathic characteristics (Ohtori *et al.,* 2012), and similarly, rodent models of osteoarthritis can be characterized by a neuropathic component leading to cutaneous sensitization outside the injured area which is sensitive to pregabalin inhibition (Rahman *et al.,* 2009; Thakur *et al.,* 2012). In contrast, animal models of osteoarthritis lacking neuropathic characteristics are not sensitive to pregabalin anti‐nociception (Thakur *et al.,* 2012). Correspondingly, there is some evidence that patients reporting pain using neuropathic descriptors respond positively to combination therapy with pregabalin and advocates the benefits of stratification of patients (Ohtori *et al.,* 2013). A complex interplay of inflammatory, neuropathic and disease‐specific components leads to pain chronicity in a rat model of cancer‐induced bone pain, with one consequence being a phenotypic change in the ratio of WDR‐to‐nociceptive specific (NS) neurones in the superficial dorsal horn. Chronic gabapentin not only inhibits enhanced frequencies of firing in this model but normalizes the ratio of neuronal response profiles (Donovan‐Rodriguez *et al.,* 2005). In comparison, spinal nerve‐ligated rats do not display similar increases in neuronal firing frequency in the dorsal horn. Instead, an increase in population coding is proposed to mediate hypersensitivity which subsequently converges onto thalamic relays. In the ventral posterolateral (VPL) thalamus of SNL rats, mechanical‐ and cold‐evoked responses, and to a lesser extent heat responses, of WDR and NS neurones are increased and pregabalin normalizes neuronal coding in a modality selective manner (mechanical and heat) at intensities evoking elevated responses (Patel and Dickenson, 2016). Mechanically evoked responses (brush and punctate stimuli) are inhibited to a greater extent compared with heat stimuli, and this corresponds relatively well with patient psychophysics (Attal *et al.,* 1998), though the contrasting reversal of cold hypersensitivity observed in this latter study may relate to the chronic dosing regimen.

In healthy volunteers, gabapentin is ineffective against temporal summation of pain, and similarly with the analogous process in rats, electrically evoked wind‐up of deep dorsal horn neurones is unaffected (Arendt‐Nielsen *et al.,* 2011). In contrast, in a surrogate model of central sensitization, gabapentin inhibits temporal summation (Arendt‐Nielsen *et al.,* 2007), and there is some evidence that wind‐up is inhibited in animal models where central sensitization is likely to be present such as in cancer‐induced bone pain and after sciatic nerve injury, though surprisingly not after spinal nerve injury (Donovan‐Rodriguez *et al.,* 2005; Curros‐Criado and Herrero, 2007; Bee and Dickenson, 2008; Ding *et al.,* 2014). Wind‐up is a short‐term process reflecting activity‐dependent increases in neuronal excitability and can lead to features shared with central sensitization such as expansion of receptive field size, inducing wind‐up at lower frequencies and overlapping pharmacological dependencies (e.g. reversal by http://www.guidetopharmacology.org/GRAC/FamilyDisplayForward?familyId=75 antagonists) (Li *et al.,* 1999). Thus, wind‐up could act as a read out of sensitization state, and pharmacological agents that inhibit wind‐up may also reverse central sensitization.

When ongoing pain scores are used as a clinical endpoint, the NNT for gabapentin and pregabalin is approximately 7, although this varies across different aetiologies (Finnerup *et al.,* 2010; Finnerup *et al.,* 2015). Thus, when assuming homogeneity amongst patients, most fail to achieve adequate relief. *Post hoc* sensory profiling analysis, however, reveals that the presence of pinprick hyperalgesia is a predictor of efficacy in human immunodeficiency virus (HIV)‐induced neuropathy (Simpson *et al.,* 2010) and correlates with surrogate models of sensitization in humans and neuronal characterizations in rodents as gabapentin and pregabalin reduce mechanical hypersensitivity (Werner *et al.,* 2001; Dirks *et al.,* 2002; Segerdahl, 2006; Chizh *et al.,* 2007; Patel and Dickenson, 2016). In CPP assays, neither gabapentin nor pregabalin has rewarding properties in the absence of injury (Andrews *et al.,* 2001), whereas in neuropathic and inflammatory models, gabapentin has been demonstrated to activate reward pathways (Xie *et al.,* 2014; Griggs *et al.,* 2015; Park *et al.,* 2016), though notably this occurs at 10‐fold higher doses than required to inhibit spinal neuronal responses to evoked stimulation (Suzuki *et al.,* 2005). The mechanisms that underpin ongoing pain may be distinct from those that mediate evoked pain. The neural mechanisms by which gabapentin provides relief from ongoing pain are poorly characterized. Despite reports of reductions in spontaneous spinal neuronal activity by gabapentin (Chapman *et al.,* 1998; Suzuki and Dickenson, 2006; Dong *et al.,* 2013; Zhang *et al.,* 2013), pregabalin has no effect on aberrant spontaneous firing in the ventral posterolateral thalamus in SNL rats (Patel and Dickenson, 2016). Spontaneous and evoked activity in the right central nucleus of the amygdala is elevated following a peripheral nerve injury, which can be normalized by pregabalin, suggesting that gabapentinoids may modulate activity within corticolimbic pathways associated with affective dimensions of pain (Goncalves and Dickenson, 2012). Collectively, these data indicate that gabapentinoids poorly relieve ongoing pain in patients but are effective in reducing areas of secondary sensitization and mechanical hyperalgesia. This difference could contribute to high NNTs if these sensory modalities were not subdivided in clinical studies. Importantly, patients have pain scores well above the pain threshold. As gabapentinoids attenuate the firing of spinal and thalamic sensory neurones to noxious intensities at doses that would fully reverse behavioural hypersensitivity to lower intensity stimuli, these may act as a better predictor of their anti‐hyperalgesic effect.

### Collapsin response mediator protein 2 (CRMP2)

Even though gabapentinoids can be effective for the management of pain, they are not without adverse effects such as ataxia, nausea, somnolence and dizziness. Thus, new approaches to target channel trafficking have been explored. Ca_V_2.2 channels present in the presynaptic terminals of primary afferent fibres forms part of a large complex of proteins and other molecules including a regulatory protein CRMP2. This protein has been shown to enhance membrane trafficking of Ca_v_2.2 channels, and CRMP2 knockdown reduces calcium currents and transmitter release, suggesting that Ca_v_2.2 channels require the presence of this protein for normal function (Chi *et al.,* 2009). CBD3 is a 15‐amino acid peptide region (ARSRLAELRGVPRGL) of CRMP2 that binds to Ca_v_2.2 channels, and a CBD3 and TAT (HIV‐1 trans‐activator of transcription) conjugated brain penetrant peptide has been shown to block the interaction of CRMP2 and Ca_v_2.2 channels (Francois‐Moutal *et al.,* 2015). Electrophysiological studies in rat spinal cord splices demonstrated that perfusion with TAT‐CBD3 reduces CGRP release (Brittain *et al.,* 2011). Unlike the gabapentinoids, TAT‐CBD3 does not display pathological state‐dependent activity and inhibits acute nociception and inflammation‐induced hypersensitivity but not nerve injury‐induced hypersensitivity (Wilson *et al.,* 2011; Francois‐Moutal *et al.,* 2015). At effective analgesic doses, TAT‐CBD3 does not produce motor impairment, paralysis or cognitive deficits in rats (Wilson *et al.,* 2011). A similar peptide, R9‐CBD3‐A6K, has no aversive or rewarding properties in sham rats but activates reward pathways in a neuropathic state (Xie *et al.,* 2016). Taking into account these results, TAT‐CBD3 could be a potential drug for the treatment of chronic pain, though the poor bioavailability and short half‐life of peptide agents limits their usefulness.

## Targeting α1 VGCC subunits

### Activation state‐independent channel blockers

**Figure 2:**
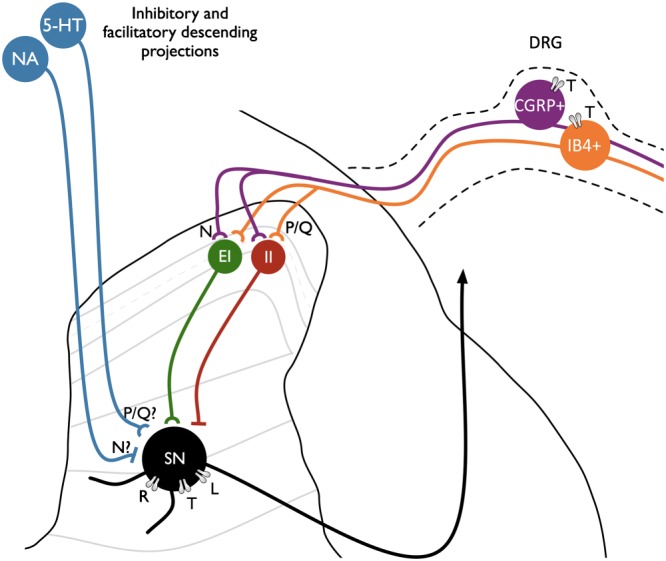
Expression of VGCCs in the dorsal horn. Ca_V_2.1 (P/Q‐type) and Ca_V_2.2 (N‐type) channels are largely expressed in distinct populations of primary afferents with the latter associated with peptidergic transmitters (Westenbroek *et al.,* 1998). Ca_V_3.2 (T‐type) channels are expressed in CGRP+ and IB4+ DRG neurones and also in cutaneous nerve endings (Rose *et al.,* 2013). Both populations synapse with markers of excitatory (EI) and inhibitory (II) interneurons which subsequently converge onto second‐order sensory neurones (SN). The effects of state‐independent blockers on reducing excitatory transmission from primary afferents may be countered by opposing effects in reducing inhibitory transmitter release; for example, Ca_V_2.2 channels have been shown to mediate noradrenaline release from sympathetic neurones, though it is unclear precisely how monoamines are released in the dorsal horn. L‐type (Ca_V_1) and T‐type (Ca_V_3) channels influence post‐synaptic excitability; neuroplasticity in L‐type expression after injury may influence neuronal sensitization (Radwani *et al.,* 2016), whereas spinally expressed T‐type channels, unlike peripherally expressed channels, are not thought to have a pathophysiological role in neuropathy. R‐type (Ca_V_2.3) channels, however, have been implicated in neuronal hyperexcitability in SNL rats (Matthews *et al.,* 2007).

http://www.guidetopharmacology.org/GRAC/FamilyIntroductionForward?familyId=80 (L‐type), http://www.guidetopharmacology.org/GRAC/FamilyIntroductionForward?familyId=80 (P/Q‐type), Ca_V_2.2 (N‐type), http://www.guidetopharmacology.org/GRAC/FamilyIntroductionForward?familyId=80 (R‐type) and http://www.guidetopharmacology.org/GRAC/FamilyIntroductionForward?familyId=80 (T‐type) channels are ubiquitously distributed in the central and the peripheral nervous system. In the dorsal horn, Ca_V_2.1 and Ca_V_2.2 channels are predominantly localized in the presynaptic terminals of largely non‐overlapping populations of primary afferent fibres, whereas Ca_V_1 and Ca_V_2.3 channels are more commonly associated with somata and dendrites (Westenbroek *et al.,* 1998) (Figure 2). Ca_V_2.1 and Ca_V_2.2 channels mediate release of neurotransmitters such as http://www.guidetopharmacology.org/GRAC/LigandDisplayForward?ligandId=1369, http://www.guidetopharmacology.org/GRAC/LigandDisplayForward?ligandId=2098 and http://www.guidetopharmacology.org/GRAC/LigandDisplayForward?ligandId=695 from primary afferent fibres causing the activation of second‐order neurones (Santicioli *et al.,* 1992; Luebke *et al.,* 1993; Terashima *et al.,* 2013). Ca_V_1 and Ca_V_2.3 channels on the other hand are associated with post‐synaptic excitability; the former appears to underlie the ability of a subset of dorsal horn neurones to mount significant trains of post‐discharge (Morisset and Nagy, 1999), whereas the genetic ablation of the latter demonstrates little role in basal nociception but a reduction of inflammatory hypersensitivity (Saegusa *et al.,* 2000).

#### ω‐agatoxin IVA

In trigeminal second‐order neurones, http://www.guidetopharmacology.org/GRAC/LigandDisplayForward?ligandId=2529 has a small inhibitory effect on cold‐evoked responses but enhances spontaneous activity in a http://www.guidetopharmacology.org/GRAC/FamilyDisplayForward?familyId=72 receptor‐sensitive manner (Ebersberger *et al.,* 2004), consistent with a projection to both excitatory and inhibitory interneurones in the dorsal horn (Westenbroek *et al.,* 1998). Spinally application of the Ca_V_2.1 channel blocker, ω‐agatoxin IVA, has a low inhibitory effect on spinal WDR neuronal responses to innocuous and noxious mechanical stimulation in normal rats (Nebe *et al.,* 1997; Matthews and Dickenson, 2001a) and has a similar magnitude of effect in SNL rats (Matthews and Dickenson, 2001a). In contrast, the potency of ω‐agatoxin IVA is enhanced after an acute inflammatory insult (Nebe *et al.,* 1997) and supports a role of Ca_V_2.1 channels in the development of sensitization but argues against a pathophysiological role in a chronic neuropathic state.

#### Ca_V_3 channel blockers

Likewise, in uninjured conditions, the Ca_V_3 channel blocker http://www.guidetopharmacology.org/GRAC/LigandDisplayForward?ligandId=7182 modestly inhibits WDR responses to mechanical and heat stimulation and exhibits similar potency after peripheral nerve injury (Matthews and Dickenson, 2001b), again arguing against a pathophysiological role of spinal Ca_V_3 channels at least. Unlike Ca_V_2.1 and Ca_V_2.2 channels, Ca_V_3 channels activate at voltages close to resting membrane potential and the gating properties permit regulation of oscillations and burst firing. Intrathalamic delivery of http://www.guidetopharmacology.org/GRAC/LigandDisplayForward?ligandId=7718, an activation state‐dependent Ca_V_3.2 blocker, reduces burst firing in the VPL of neuropathic rats, an observation that correlates with the establishment of CPP when Z944 is dosed systemically (LeBlanc *et al.,* 2016). Peripherally expressed Ca_V_3.2 channels may also be important as they modulate excitability of a subset of mechanoreceptors (Shin *et al.,* 2003), and Ca_V_3‐mediated currents are enhanced in injured dorsal root ganglion (DRG) neurones (Jagodic *et al.,* 2008). In SNL rats, the peripherally acting state‐dependent blocker, http://www.guidetopharmacology.org/GRAC/LigandDisplayForward?ligandId=7721, reduces spinal WDR neuronal responses to low intensity mechanical stimulation (Jarvis *et al.,* 2014).

#### ω‐conotoxin GVIA

Studies have shown that, α1B (Ca_V_2.2) deficient mice have a normal life span and normal behaviour, however have impaired basal nociception and development of neuropathy (Hatakeyama *et al.,* 2001; Saegusa *et al.,* 2001). The role this channel plays in the transmission of nociceptive stimuli is further supported by electrophysiological studies demonstrating that http://www.guidetopharmacology.org/GRAC/LigandDisplayForward?ligandId=2535 inhibits mechanical‐ and heat‐evoked responses and wind‐up of spinal WDR neurones consistent with a role in mediating peptide release and consequentially post‐synaptic hyperexcitability. Dural afferents also express Ca_V_2.2 channels, and blockade reduces cold‐evoked and spontaneous activity of trigeminal nucleus neurones (Ebersberger *et al.,* 2004). The increased potency of ω‐conotoxin GVIA after SNL indicates injury‐induced neuroplastic changes in channel expression and/or regulation in the dorsal horn (Matthews and Dickenson, 2001a). In contrast, no change in the effect size of ω‐conotoxin GVIA is observed on spinal WDR neurones innervating the knee following knee inflammation (Neugebauer *et al.,* 1996). Intrathecal ω‐conotoxin MVIIA can induce CPP in SNL rats demonstrating an alleviation of ongoing pain and correlates well with clinical observations (King *et al.,* 2009).

ω‐Conotoxins are small peptides derived from the venom of marine cone snails and block a series of ion channels. These peptides are thought to act by physical occlusion of the α1 ion conducing pore (Bourinet and Zamponi, 2016). In animal models, conotoxins such as GVIA, MVIIA and http://www.guidetopharmacology.org/GRAC/LigandDisplayForward?ligandId=2534 have analgesic properties against inflammatory and neuropathic pain; however, they have also been shown to have a series of negative side effects in patients such as dizziness, nausea, nystagmus, gait abnormality, urinary retention, memory impairment and hallucinations. Some of them such as GVIA have irreversible effects once they bind to the pore, which complicates its usage. MVIIA, also known as ziconotide has been the first ω‐conotoxin to enter clinical trials and was approved in 2004 by the FDA, for the management of chronic pain. Initial clinical trials conducted in patients with chronic pain, cancer‐related or AIDS‐related neuropathy refractory to opioid treatment reported improvement of VAS (visual analogue scale) scores (from a mean baseline of >70/100) by 14 to 53%, while the proportion of responders ranged from 16 to 50% (Sanford, 2013). The dosage was often titrated to tolerable levels which may account for some of the variability in effect size. Even though ziconotide does not produce tolerance or addiction like http://www.guidetopharmacology.org/GRAC/LigandDisplayForward?ligandId=1627 does, it has adverse neurological side effects that need to be taken into account before prescribing the drug to a patient. In addition, its large amino acid sequence has some limitations. For instance, manufacturing the drug is challenging and expensive. Due to its large size, ziconotide poorly crosses the brain–blood barrier, thus needs to be delivered straight into the spinal cord or in the surrounding cerebrospinal fluid for the treatment of pain.

Due to the limitations of ziconotide, there is a need to develop newer blood–brain barrier penetrable small molecule calcium channel blockers with fewer adverse effects as well as drugs that are orally available. Several new activation state‐dependent blockers have been developed and tested in animal models with some progressing to clinical trials.

### Activation state‐dependent channel blockers

#### ZC88

ZC88 ((*N*‐[1‐(2,3‐dimethoxyphenyl)‐4‐methyl‐3‐pentene‐1‐yl]‐1‐(5‐bromo‐furfuryl)‐1‐piperidyl‐4‐amine dihydrochloride)) is a non‐peptide, state‐dependent, blocker of Ca_V_2.2 channels and does not affect calcium currents mediated by Ca_V_1, Ca_V_2.1 or Ca_V_2.3 channels in cultured DRG neurones (Zhang *et al.,* 2015). Behavioural studies in a rat chronic constriction injury model of neuropathic pain show a dose‐dependent decrease of mechanical hypersensitivity after oral administration of ZC88 (Meng *et al.,* 2008). However, ZC88 administration has no effect on acute nociception in normal mice in the hot plate test but enhances morphine analgesia. This synergy may arise as opiates can inhibit Ca_V_2.2 via G_i/o_ protein‐coupled http://www.guidetopharmacology.org/GRAC/ObjectDisplayForward?objectId=319 receptors. As chronic treatment with morphine is associated with tolerance and addiction, Meng *et al.,* (2008) tested the effects of ZC88 in animals that had been chronically treated with morphine. In isolation, ZC88 does not produce CPP in uninjured mice. However, in combination with morphine, ZC88 reverses tolerance to chronic morphine in the hot plate test without affecting morphine‐induced CPP. These results are promising, as ZC88 could be a potential drug against neuropathic pain that could be administered together with morphine or used alone.

#### A‐1264087

A‐1264087 (*N* ^2^‐methyl‐*N*‐{(3a*R*,4*S*,6a*S*)‐2‐[4‐(trifluoromethyl)phenyl]octahydrocyclopenta[*c*]pyrrol‐4‐yl}‐l‐leucinamide) is a state‐dependent blocker of Ca_V_2.1, Ca_V_2.2 and Ca_V_3 channels. This drug has been shown to block Ca_V_2.2 channels in cultured rat DRG neurones as well as to reduce spontaneous and mechanical‐evoked activity of WDR neurones in SNL animals. Systemic administration reduces mechanically evoked neuronal responses to low‐intensity von Frey filaments as well as Aδ‐fibre and C‐fibre‐evoked responses to electrical stimulation and subsequent wind‐up, selectively in neuropathic rats (Xu *et al.,* 2014). Correspondingly, using withdrawal reflexes as an endpoint, A‐1264087 after oral administration in SNL rats and the iodoacetate model of osteoarthritis attenuates mechanical hypersensitivity (Xu *et al.,* 2014; Zhu *et al.,* 2014). Both of these studies report a decrease in neuronal responses to mechanical stimulation. However, neither of these studies assessed changes in neuronal responses to other modalities or to more noxious intensities of stimulation where C‐fibres are recruited. Thus, it would be interesting to further analyse the effects of A‐1264087 on high intensities of stimulation. Interestingly, in SNL rats, spontaneous spinal neuronal activity is reduced by A‐1264087 when dosed spinally but not when delivered directly to the receptive field (Xu *et al.,* 2014). It would be of value to investigate the effects in operant‐based assays to examine whether A‐1264087 alleviates ongoing as well as evoked pain.

#### TROX‐1

N‐triazole oxindole (http://www.guidetopharmacology.org/GRAC/LigandDisplayForward?ligandId=7766) is an orally bioavailable, blood–brain barrier penetrating, state‐dependent blocker of the Ca_V_2 family. In cultured cell systems, blocking channels under depolarized conditions, TROX‐1 has similar potency across the Ca_V_2 family, thus acting on Ca_V_2.1, Ca_V_2.2 and Ca_V_2.3 channels (Swensen *et al.,* 2012). Behavioural studies demonstrate that in animal models of acute inflammatory pain, oral administration of TROX‐1 reverses behavioural hypersensitivity (Abbadie *et al.,* 2010). However, in Ca_V_2.2 knockout mice, TROX‐1 is ineffective, suggesting that TROX‐1 primarily mediates its effects through Ca_V_2.2 channels in inflammatory conditions, despite evidence that Ca_V_2.3 channels also contribute to spinal neuronal hyperexcitability after injury (Matthews *et al.,* 2007).

TROX‐1 is also anti‐nociceptive in more severe chronic models of pain. Behavioural assessments have also shown that TROX‐1, given by oral, subcutaneous and intrathecal routes, increases paw withdraw thresholds in SNL rats in a dose‐dependent manner with no effect in sham‐operated animals (Abbadie *et al.,* 2010; Patel *et al.,* 2015). In concordance with these behavioural results, *in vivo* electrophysiological recordings in SNL rats showed a reduction of neuronal‐evoked responses to low intensity and supra‐threshold mechanical stimulation with von Frey filaments after spinal and systemic administration of TROX‐1 (Patel *et al.,* 2015). This effect was modality selective; brush‐, heat‐ and cold‐evoked responses were unaffected by TROX‐1 in the SNL model. Unlike ω‐conotoxin GVIA, TROX‐1 had no effect on neuronal wind‐up. This is despite displaying use dependency in cell lines *in vitro* using comparable stimulus parameters*,* which highlights the importance of physiological assays when assessing drug actions.

As described earlier, rodent models of osteoarthritis can share common features with neuropathy resulting from peripheral nerve damage, for example, concurrent time‐dependent increases in descending facilitatory drive and loss of descending noradrenergic inhibition (Rahman *et al.,* 2009; Burnham and Dickenson, 2013). Hence, the effects of TROX‐1 were additionally examined in the rat intra‐articular iodoacetate model of osteoarthritis, where treatment with TROX‐1 reversed behavioural hypersensitivity and weight‐bearing behaviour (Abbadie *et al.,* 2010). *In vivo* electrophysiological studies have also assessed the efficacy of TROX‐1 in the iodoacetate model of osteoarthritis. Spinal administration of TROX‐1 inhibits evoked WDR neuronal responses to dynamic brush, heat and mechanical stimulation in a dose‐dependent manner (Rahman *et al.,* 2015). Systemic administration of this drug reduces supra‐threshold mechanically evoked responses to von Frey filaments, but does not affect heat and dynamic brush responses, perhaps because a more local administration of the drug (spinal administration) allows for attenuation of these modalities. In sham animals, TROX‐1 does not affect the neuronal responses after evoked stimulation. As observed in SNL rats, TROX‐1 does not reduce wind‐up in arthritic rats; the differential effects of A‐1264087 and TROX‐1 on wind‐up may relate to the selectivity profiles of the drugs. Inactivation of the rostral ventromedial medulla with lidocaine in osteoarthritic rats induces CPP and reveals the presence of ongoing pain (Havelin *et al.,* 2016); as with A‐1264087 and ZC88, it would be of interest to examine whether TROX‐1 modulates affective dimensions of pain. Together, these results suggest that the use of state‐dependent blockers for the treatment of osteoarthritic pain with neuropathic features could be a good alternative to current treatments. The differences between the actions of TROX‐1 in different pain conditions might be due to differences in state‐dependency of the channel such as the depolarizing effect of ionotropic facilitatory http://www.guidetopharmacology.org/GRAC/FamilyDisplayForward?familyId=68 receptors on pre‐synaptic terminals, altered G‐protein‐mediated regulation by http://www.guidetopharmacology.org/GRAC/FamilyDisplayForward?familyId=4, GABA‐ and http://www.guidetopharmacology.org/GRAC/FamilyDisplayForward?familyId=1, or changes in the up‐regulation of C‐terminus splice variants of calcium channels which might contribute to changes in biophysical properties of the channel.

When new drugs are developed, the implications of alternative splicing and the effects it has on channel activity are rarely considered. Multiple variants are predicted to exist, but in particular, mutually exclusive exons e37a and e37b of Ca_V_2.2 are highly enriched in DRG neurones. These exons confer different properties to the channel, for instance e37a‐Ca_V_2.2 exhibits larger calcium currents, increased channel open time and slower inactivation than e37b‐Ca_V_2.2 (Castiglioni *et al.,* 2006). VGCCs with these gating properties could be more amenable to block by state‐dependent blockers. However, the role of splice variants in the pathophysiology of neuropathic pain is complex. RNA knockdown has shown that the e37a isoform mediates the transmission of mild mechanical stimulation and heat stimulation and prevents the development of mechanical hypersensitivity in neuropathic and inflammatory models (Altier *et al.,* 2007). Paradoxically, following peripheral nerve injury, there is a decrease in the expression of the e37a isoform (Altier *et al.,* 2007). Neuroplastic shifts in splice variant expression with differences in drug sensitivity and sensitivity to G‐proteins could affect the efficacy of analgesics after injury, including morphine (Jiang *et al.,* 2013), but also, the gabapentinoids as the predominant α_2_δ‐1 splice variant up‐regulated after nerve injury has reduced affinity for gabapentin (Lana *et al.,* 2014).

## Are calcium channels a good target for chronic pain?

**Figure 3:**
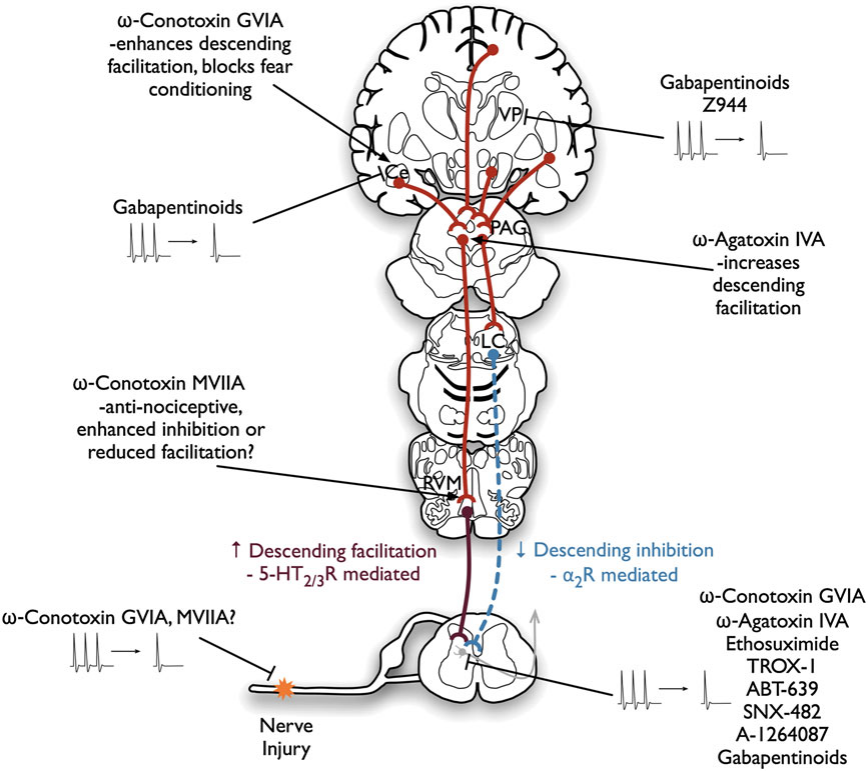
Effects of calcium channel modulators in the pain neuraxis. There are conflicting reports as to whether Ca_V_2.2 channels contribute to ectopic firing in injured primary afferent fibres (Chaplan *et al.,* 1994; Xiao and Bennett, 1995). However, numerous studies demonstrate that state‐dependent and ‐independent blockers can reduce spinal neuronal hyperexcitability when given systemically or spinally. In addition, systemic pregabalin reduces neuronal firing in the amygdala and VPL. Neuropathy can be associated with diminished descending noradrenergic inhibitory control of pain and enhanced serotonergic facilitatory drive. Both Ca_V_2.1 and Ca_V_2.2 channels have complex roles in descending modulation and exert pro‐ and anti‐nociceptive actions when micro‐injected into different regions (Knight *et al.,* 2002; Finn *et al.,* 2003; Urban *et al.,* 2005). However, state‐dependent blockers targeting high‐frequency neuronal firing may avoid the confounding actions of state‐independent blockers (Ce, central nucleus of amygdala; LC, locus coeruleus; PAG, periaqueductal gray; RVM, rostral ventromedial medulla; VP, ventral posterior thalamus).

Due to their critical role in transmitter release, calcium channels are regarded as an important target in preventing sensory transmission. However, the ubiquitous nature of expression and multitude of processes controlled by calcium channels has complicated the use of blockers for chronic pain (reviewed in detail by Zamponi *et al.,* 2015). Although spinally delivered state‐independent blockers can reduce behavioural and spinal hyperexcitability, micro‐injection of peptide blockers into brainstem regions mediating descending control of pain reveals complex pro‐ and anti‐nociceptive roles of calcium channels and could confound usage of blockers via a systemic route (Figure 3). Targeting nociceptor specific splice variants to improve specificity and reduce side effects seems unlikely to yield positive results given the notorious difficulty in identifying selective low MW blockers of ion channels, let alone selective blockers for splice variants that differ only by small sequences. In theory, state‐dependent blockers would circumvent the confounds of blocking calcium channels by targeting high‐frequency neuronal firing associated with neuropathic pain. In this aspect, compounds such as CNV2197944 (Ca_v_2.2), ABT‐639 (Ca_v_3.2) and Z160 (Ca_v_2.2), which exhibit activation state dependency, were generally well tolerated in phase I clinical trials. Unfortunately, Z160 (NCT1655849) and ABT‐639 (NCT01345045) failed to show analgesic efficacy in phase II trials, whereas results for CNV2197944 have not been reported yet (NCT01848730 and NCT01893125).

Z160 is anti‐nociceptive in a range of animal models when examined against withdrawal reflexes (Lee and Snutch, 2013) but failed in phase II trials with ongoing pain as the primary endpoint. It is conceivable that a failure to consider heterogeneity in patients during trial design led to an overall negative outcome. Drawing parallels with anticonvulsant sodium channel blockers which are more efficacious in patients with the irritable nociceptor phenotype, that is, where peripheral afferents are hyperexcitable and patients have evoked hypersensitivity, a state‐dependent calcium channel blocker could also be efficacious in this subgroup (Bouhassira and Attal, 2016; Baron *et al.,* 2017). Likewise, ABT‐639 failed to ameliorate ongoing pain in diabetic polyneuropathy patients (Ziegler *et al.,* 2015). Interestingly, ABT‐639 has no effect on spontaneous firing of WDR neurones in SNL rats but inhibits mechanically evoked firing (Jarvis *et al.,* 2014). Diabetic polyneuropathy is most commonly associated with the ‘sensory loss’ phenotype, whereas a smaller proportion are classified under the ‘mechanical hyperalgesia’ phenotype (Baron *et al.,* 2017); it is possible that ABT‐639 may have had higher efficacy in this subgroup in this trial (Ziegler *et al.,* 2015). The inability of ABT‐639 to cross the blood–brain barrier could also have contributed to an overall negative result.

## Conclusion

Despite disappointing clinical trials to date, it would be premature to consign calcium channels as a chronic pain target to the history books. A progressive approach to clinical trial design will be key to the success of future therapeutic approaches. Therapeutic agents displaying pathological state dependency would be preferable to avoid perturbing normal sensory function and to limit side effects. Poly‐pharmacy could be an alternative approach as gabapentin and ω‐conotoxins have demonstrated synergy with morphine (Matthews and Dickenson, 2002; Kolosov *et al.,* 2011). It would be of interest to examine whether state‐dependent calcium channel blockers also exhibit similar synergistic effects. In the short term, patients receiving intrathecal ziconotide may benefit from improved analgesia and fewer side effects with combination therapy.

### Nomenclature of targets and ligands

Key protein targets and ligands in this article are hyperlinked to corresponding entries in http://www.guidetopharmacology.org, the common portal for data from the IUPHAR/BPS Guide to PHARMACOLOGY (Southan *et al.,* 2016), and are permanently archived in the Concise Guide to PHARMACOLOGY 2015/16 (Alexander *et al.,* 2015a,b).

## Conflict of interest

A.H.D has received research funding from Grünenthal GmbH.
